# Anti-inflammatory therapy for tendinopathy using *Il1rn* mRNA encapsulated in SM102 lipid nanoparticles

**DOI:** 10.3389/fbioe.2025.1641236

**Published:** 2025-08-12

**Authors:** Yuan Zhang, Xu Li, Hao Li, Ruiyang Zhang, Ti Zhang, Talante Juma, Yongfei Zhou, Quanyi Guo, Hui Zhao, Yongping Cao

**Affiliations:** ^1^ Department of Orthopedics, Peking University First Hospital, Beijing, China; ^2^ Key Laboratory for Regenerative Medicine of the Ministry of Education of China, School of Biomedical Sciences, Faculty of Medicine, The Chinese University of Hong Kong, Shatin, Hong Kong SAR, China; ^3^ National Center for Orthopaedics, Beijing Jishuitan Hospital, Capital Medical University, Beijing, China; ^4^ Beijing Key Lab of Regenerative Medicine in Orthopedics, Key Laboratory of Musculoskeletal Trauma & War Injuries PLA, Institute of Orthopedics, The First Medical Center, Chinese PLA General Hospital, Beijing, China; ^5^ Department of Orthopedics, Peking University Third Hospital, Beijing, China; ^6^ Institute of Hemu Biotechnology, Beijing Hemu Biotechnology Co., Ltd., Beijing, China

**Keywords:** tendinopathy, mRNA therapy, SM102 lipid nanoparticles, interleukin-1 receptor antagonist, anti-inflammatory therapy

## Abstract

Tendinopathy treatment is hindered by persistent inflammation and irreversible matrix degradation, with current therapies offering transient symptom relief without addressing disease progression. Here, we developed an mRNA-based anti-inflammatory strategy utilizing SM102 lipid nanoparticles (LNPs) to deliver interleukin-1 receptor antagonist (*Il1rn*) mRNA for tendon repair. SM102-LNPs demonstrated efficient transfection of primary tendon stem cells, sustaining IL-1RA protein expression for over 72 h and neutralizing IL-1β-induced inflammatory cascades. *In vitro*, IL-1RA suppressed pro-inflammatory cytokines (TNF-α, IL-6, iNOS), restored collagen I/III balance, and enhanced cell migration. In collagenase-induced tendinopathy mice, a single SM102-*Il1rn* mRNA injection attenuated inflammation, reduced MMP1/13 expression, and improved collagen alignment within 1 week. By 4 weeks, treated tendons exhibited functional recovery with normalized gait patterns. Transcriptomics revealed dual modulation of IL-1 signaling and extracellular matrix (ECM) remodeling pathways, alongside macrophage polarization and oxidative stress regulation. Systemic safety was confirmed by unaltered serum biomarkers and organ histology. This SM102-*Il1rn* mRNA therapy enables spatiotemporally controlled anti-inflammatory therapy, providing a promising non-surgical solution for refractory tendinopathies. Its adaptable design allows expansion to other regenerative targets, advancing precision treatment for musculoskeletal degeneration.

## 1 Introduction

Tendinopathy, as a common degenerative disease in the musculoskeletal system, has a high incidence rate among professional athletes and the elderly population (Kwan et al., 2023; Loiacono et al., 2019). Its pathological features are characterized by abnormal activation of pro-inflammatory factors, accelerated degradation of type I collagen due to upregulation of matrix metalloproteinases, and elevated expression of fibrosis-related genes (Loiacono et al., 2019; Millar et al., 2021; Riley, 2008; Morita et al., 2017). Disordered collagen structure and loss of biomechanical function of the tendon triggered clinical symptoms such as pain and swelling (Millar et al., 2021). The main clinical treatments for tendinopathy include NSAIDs, glucocorticoids, and physical therapy, but this therapy can only provide short-term symptom relief and cannot reverse the degenerative process (Andres and Murrell, 2008; Adjei-Sowah et al., 2024). Moreover, pieces of evidence supported that the use of NSAIDs and glucocorticoids may elevate the risk of tendon rupture and toxic tendinopathy (Coombes et al., 2010; Canosa-Carro et al., 2022).

In recent years, the regulation of the inflammatory microenvironment has become an important direction in tendon repair research (Chisari et al., 2020). The interleukin-1 receptor antagonist (IL-1RA), as an endogenous anti-inflammatory protein, blocks the IL-1β signaling pathway by competitively binding to IL-1R1 receptors (Arend, 1993; Gabay et al., 2010). Clinical and pre-clinical studies have shown the good safety and efficacy of recombinant IL-1RA (commercially known as “anakinra”) in treating osteoarthritis (Iqbal and Fleischmann, 2007; Pelletier et al., 1997; Nuki et al., 2002; Fleischmann et al., 2006). In the field of tendon diseases, local injection of recombinant IL-1RA protein can significantly improve the pathological process of rat tendonitis models (Berkoff et al., 2016; Eskildsen et al., 2019). However, this protein has limitations such as a short half-life (4–6 h), poor stability, and susceptibility to degradation, necessitating frequent injections (Nuki et al., 2002; Fleischmann et al., 2006; Tarighi et al., 2021; Dahlén et al., 2008; Yang et al., 2019).

Recent advancements in regenerative approaches have highlighted the potential of nucleic acid-based interventions, with mRNA delivery platforms demonstrating particular promise as clinically applicable treatment strategies (Balmayor, 2022). Compared with traditional DNA therapy, mRNA can be translated and expressed without entering the cell nucleus, fundamentally avoiding the risk of genome integration (Hou et al., 2021; Sahin et al., 2014). Its time-limited expression feature (usually lasting for several days) and dose controllability are particularly suitable for the treatment of inflammatory diseases that require precise regulation (Hou et al., 2021; Sahin et al., 2014; Weissman, 2015). In selecting delivery systems, lipid nanoparticles (LNP) stand out due to their mature preparation process and verified safety (Sahin et al., 2014). The core component, ionizable lipid SM102, promotes endosomal escape through a pH-responsive mechanism, and its delivery efficiency has been confirmed in the muscle tissue transfection of the COVID-19 vaccine (Schoenmaker et al., 2021; Zhang et al., 2023). However, the potential application of this delivery system in tendon tissues still needs to be verified.

Our study was the first to demonstrate that mRNA delivery mediated by SM102-LNP can be used to treat tendinitis. *In vitro* experiments have shown that SM102-LNP can efficiently transfect primary tendon stem cells and maintain the expression of target genes for at least 72 h. Meanwhile, tendon stem cells transfected with SM102- *Il1rn* mRNA can synthesize and secrete bioactive IL-1RA and provide a protective effect on tendon stem cells under IL-1β stimulation. *In vivo* experiments have proved that a single injection of SM102 LNPs/mRNA complex can successfully deliver mRNA to tendon tissue cells. Local injection of SM102- *Il1rn* mRNA at the tendon site could inhibit inflammation, accelerate the repair of tendon tissue structure and function, and exhibit good biological safety. The SM102 LNPs delivery system has the advantage of modular design. By replacing the coding sequence, it can be flexibly expanded to deliver other anti-inflammatory factors such as IL-10 and TGF-β inhibitors. In the future, by optimizing the tendon targeting of LNP, this strategy is expected to provide a new non-surgical intervention scheme for refractory tendon diseases and has important clinical application prospects.

## 2 Materials and methods

### 2.1 mRNA synthesis

The mRNA (*GFP* or mouse *Il1rn*)was synthesized via *in vitro transcription* (IVT). Briefly, the coding sequence (CDS) of *EGFP* or mouse *Il1rn* was inserted into the IVT template plasmid. The CDS was flanked by a T7 promoter and a poly(A) tail sequence. The plasmid was linearized through restriction enzyme digestion, and the mRNA was transcribed. After purification, the size and purity of the synthesized mRNA were analyzed by denaturing agarose gel electrophoresis. The concentration of mRNA was determined by Nanodrop (A260/A280 ratio ∼2.0).

### 2.2 SM102 lipid nanoparticle (LNP) formulation and mRNA encapsulation

The SM102-LNP formulation was prepared as previously described (Hassett et al., 2019). Briefly, ionizable lipid (SM102), Phospholipid (DSPC) Cholesterol, and PEGylated lipid (DMG-PEG2000) were dissolved in ethanol with a mass ratio of 50:10:38.5:1.5. The LNPs containing mRNA were assembled via the microfluidic device. RNA was dissolved in citrate buffer (pH 4.0, 50 mM) and the aqueous phase and organic phase were combined at a 3:1 volumetric ratio, achieving rapid mixing in a staggered herringbone micromixer. The SM102-LNP/mRNA complexes were diluted in RNase-free PBS buffer and purified using a cutoff membrane (30 kDa). The final concentration of mRNA was 60 μg/mL and the sucrose in PBS was 10%. Efficiency was determined by Quant-iT RiboGreen RNA Assay Kit according to the user’s instructions. Particle Size and Polydispersity Index (PDI, DLS) were measured by Dynamic light scattering. The morphology of SM102 LNPs-*Il1rn* was observed using transmission electron microscopy (JEOL).

### 2.3 Cell culture and transfection

Mouse primary tendon stem cells (purchased from Wuhan Pricella Biotechnology Co., Ltd., CP-M176) and HEK293T (purchased from Bkmam Holdings Co., Ltd., CL0005) were cultured in complete growth medium (DMEM supplemented with 10% FBS and 1% penicillin/streptomycin) at 37°C with 5% CO_2_. Cells were seeded at an appropriate density. On the 2^nd^ day, the cells were incubated with SM102 LNPs-*GFP* mRNA. The GFP signals were checked under fluorescent microscopy at the indicated time point post-transfection. For flow cytometry, at 48 h post-transfection, cells were trypsinized, and the GFP^+^ cell populations were quantified. Untransfected cells served as negative controls.

### 2.4 IL-1RA expression analysis

Mouse primary tendon stem cells or HEK293T cells were seeded cells in 12-well plates (1 × 10^5^ cells/well) and were incubated with SM102 LNPs- *l1rn* mRNA with indicated dosage. Supernatants were collected at the indicated time point post-transfection and IL-1RA levels were quantified by ELISA (ABclonal, RK04182) following kit instructions. The size of IL-1RA secreted by transfected cells was analyzed by Western blotting, with Recombinant Human IL-1ra Protein Standard (Abcam, ab316382) used as a positive control.

### 2.5 Quantitative PCR assay

Mouse primary tendon stem cells were seeded at an appropriate density and transfected with SM102-IL-1RA. At 24 h post transfection, the transfected cells were incubated in the presence or absence of 10 ng/mL recombinant mouse IL-1β (SinoBiological, 10139-HNAE). At 24 h post stimulation, the total RNA was extracted using Trizol for qPCR analysis. Primer sequences are listed below:

**Table:** 

Gene	Primer sequence	
GAPDH	Forward Sequence	CAT​CAC​TGC​CAC​CCA​GAA​GAC​TG
	Reverse Sequence	ATG​CCA​GTG​AGC​TTC​CCG​TTC​AG
IL-10	Forward Sequence	CGG​GAA​GAC​AAT​AAC​TGC​ACC​C
	Reverse Sequence	CGG​TTA​GCA​GTA​TGT​TGT​CCA​GC
IL-6	Forward Sequence	TAC​CAC​TTC​ACA​AGT​CGG​AGG​C
	Reverse Sequence	CTG​CAA​GTG​CAT​CAT​CGT​TGT​TC
iNOS	Forward Sequence	GAG​ACA​GGG​AAG​TCT​GAA​GCA​C
	Reverse Sequence	CCA​GCA​GTA​GTT​GCT​CCT​CTT​C

### 2.6 Immunofluorescent assay

Primary tendon stem cells were seeded in 24-well plates (2 × 10^4^ cells/well). The cells were incubated with the indicated dose of SM102 LNPs- *Il1rn* mRNA for 24 h. Then the cells with or without transfection were stimulated with 10 ng/mL recombinant mouse IL-1β (SinoBiological, 10139-HNAE) for 24 h. Then the cells were fixed in 4% paraformaldehyde for 15 min, permeabilized with 0.1% Triton X-100 for 10 min, and blocked with 5% BSA for 1 h. Subsequently, cells were incubated with anti-COL1A1 antibody (ABclonal, A24112) or anti-COL3A1 (ABclonal, A3795) at 4°C overnight. After washing, ABflo^®^ 555-conjugated Goat anti-Rabbit IgG (H + L) (ABclonal, AS058) (1:500) was added and incubated at room temperature for 1 h. Actin-Tracker Green-488 (C2201S) was then applied at room temperature for 40 min, followed by two PBS washes. Finally, DAPI (1:1000) was incubated in the dark at room temperature for 10 min. Images were captured using a confocal microscope, and fluorescence intensity is quantified with ImageJ.

### 2.7 Cell scratch assay

The primary tendon stem cells were seeded in 6-well plates, and when the fusion degree reached 90%, a scratch was made using the tip of a 200 μL pipette. Images were taken at fixed positions using an inverted microscope at the indicated time points. The migration rate was calculated as: (initial area - remaining area)/initial area × 100%.

### 2.8 Establishment of the mouse tendinitis model

**FIGURE 1:**
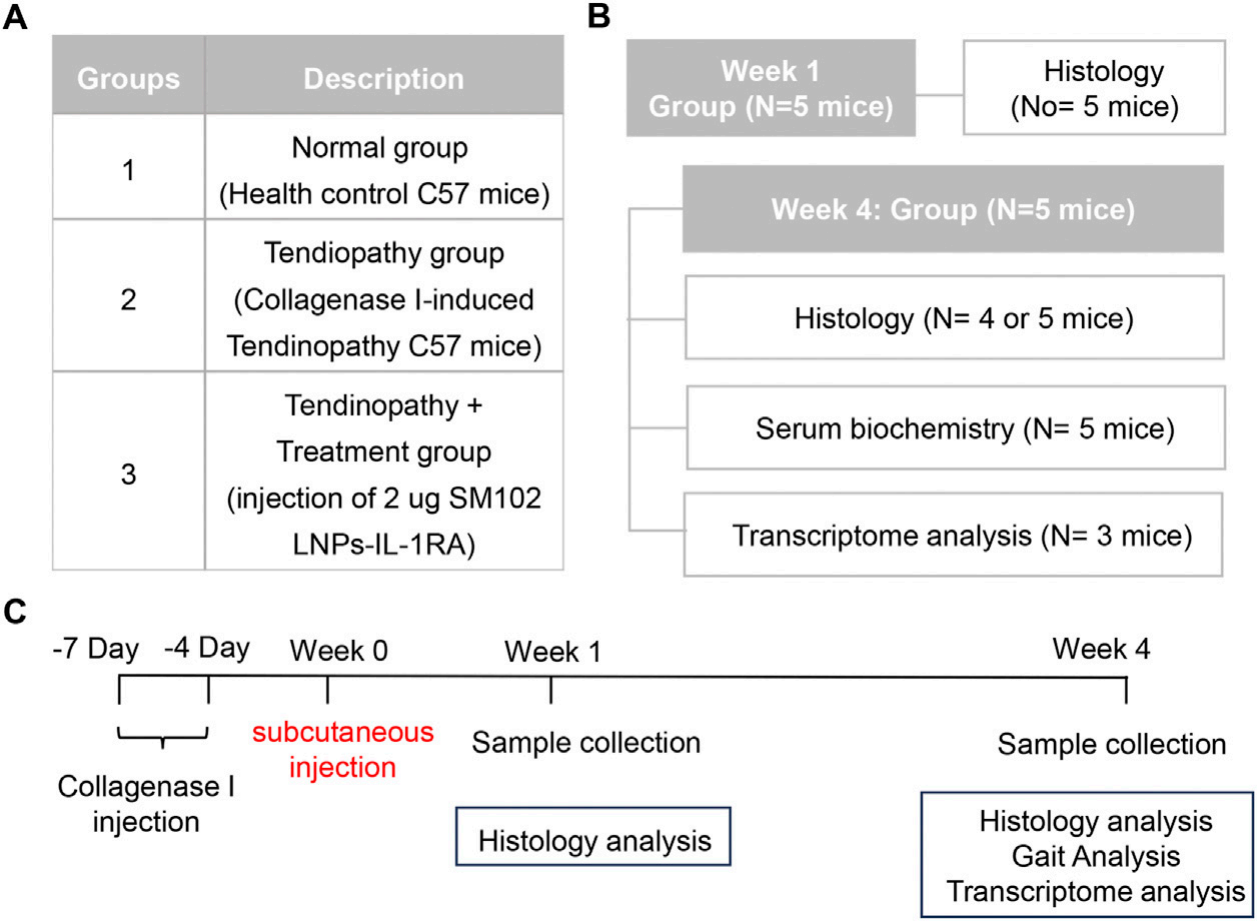
Animal experiment design. **(A)** A table illustrating group assignments and details of treatments. **(B)** A table illustrating the number of mice used for each measurement. **(C)** A diagram demonstrating treatment timelines for groups 2 and 3.

Figure 1 illustrated the group designment, details of treatments, and animal experiments timelines. To establish the mouse tendinitis model, 10-week-old male C57BL/6J mice were chosen to receive two injections of 20 μL of collagenase I (5 mg/mL, Gibco, Cat# 17100017) at the midpoint of the right Achilles tendon 7 days and 4 days before the experiment, respectively. Before experiments, the clinical assessments were performed to confirm the induction of tendinitis of the right Achilles tendon. The mice were randomly divided into the untreat group and SM102 LNPs- *Il1rn* mRNA group (2 µg of RNA per mouse). Healthy mice were used as the control group. Animals were euthanized at the respective endpoints (week 1 and week 4), and the tissue was collected for follow-up analysis. All animal trials received approval from the Institutional Animal Care and Use Committee of PLA General Hospital (SCXK No. 2019–0018).

### 2.9 *In vivo* fluorescence imaging and analysis

For *in vivo* imaging, C57BL/6 mice (20–25 g) were anesthetized with isoflurane, and 2 μg of SM102 LNPs-*luciferase* mRNA were injected into the Achilles tendon using a 30G needle. At indicated timepoints post-injection, D-luciferin (150 mg/kg in PBS) was intraperitoneally administered, followed by bioluminescence imaging of with an IVIS Spectrum system (40 s exposure, region-of-interest analysis). Immediately following live IVIS imaging, heart, liver, spleen, lung, and kidney were harvested and imaged *ex-vivo*. Data were quantified as fluorescent intensity at the region of interest.

### 2.10 Histological and immunofluorescent assessment

Tendon samples were collected and fixed in 4% paraformaldehyde overnight. After dehydration and embedding in paraffin, 5 μm paraffin sections were prepared for Hematoxylin and Eosin (H&E), Masson’s trichrome and Sirius Red staining. For immunofluorescent analysis, sections were blocked at room temperature after antigen retrieval, followed by overnight incubation at 4°C with the indicated primary antibodies [anti-COL1A1 antibody (ABclonal, A24112), anti-COL3A1 antibody (ABclonal, A3795), or anti iNOS antibody (ABclonal, A3774)] and secondary antibodies. The collagen or fluorescent positive area was quantified using ImageJ software.

### 2.11 ELISA assay on tendon tissue

Tendon tissue was collected and rinsed in cold PBS to remove blood contaminants. Then, the samples were minced into small fragments and incubated into 250 μL RIPA Lysis Buffer (Beyotime, P0038) with protease inhibitors (Beyotime, P1046). Then the mixture was centrifuged for supernatant collection. The ELISA assays for detection of IL-6 (ABclonal, RK04845) and IL-10 (ABclonal, RK04490) were performed according to the user’s instruction.

### 2.12 Gait analysis

At week 4 after treatment, gait analysis was performed using the CatWalk XT system (Noldus). In brief, the mice were placed on a glass walking platform and their walking sequences were recorded (step speed range 10–30 cm/s).

### 2.13 Transcriptome analysis on tendon tissue

At the end of the animal experiment, the tendon samples were collected and snap-frozen in liquid nitrogen. Then the frozen samples were homogenized in TRIzol reagent for total RNA extraction. RNA sequencing was performed using the Illumina sequencing platform. Reads were aligned to the mouse reference genome (mm39). Differential gene expression analysis was performed with DESeq2 (adjusted p-value <0.05, |log2 fold change| >1). Functional enrichment of pathways was analyzed using the Gene Ontology (GO) and Kyoto Encyclopedia of Genes and Genomes (KEGG) databases. Gene Set Enrichment Analysis (GSEA) was used to determine the predefined gene sets that were significantly enriched in the treatment group. All computational workflows were executed on Novogene’s online platform (NovoMagic), accessed via https://magic-plus.novogene.com/#/, and default parameters were used. Raw sequencing data were deposited in the NCBI SRA under accession number SUB15328155.

### 2.14 Biological safety evaluation

Blood serum samples were collected and immediately analyzed using an automatic biochemical analyzer (BIOBASE, BK280) to assess metabolic parameters, including alanine aminotransferase (ALT), aspartate aminotransferase (AST), total bilirubin (TBIL), direct bilirubin (DBIL), creatinine (CREA), and urea. Additionally, the heart, liver, spleen, lung, and kidney were fixed in paraffin, sectioned, and subjected to hematoxylin and eosin (H&E) staining for histological evaluation of tissue morphology.

### 2.15 Statistical analysis

All data were presented as the mean ± Standard Error of the Mean (SEM). Statistical significance was determined by one-way ANOVA or two way ANOWA with Fisher’s LSD test using GraphPad Prism 9. P *< 0.05* was considered significant.

## 3 Results

### 3.1 Assessment of the *in vitro* transfection efficiency of mRNA mediated by SM102 LNPs

**FIGURE 2:**
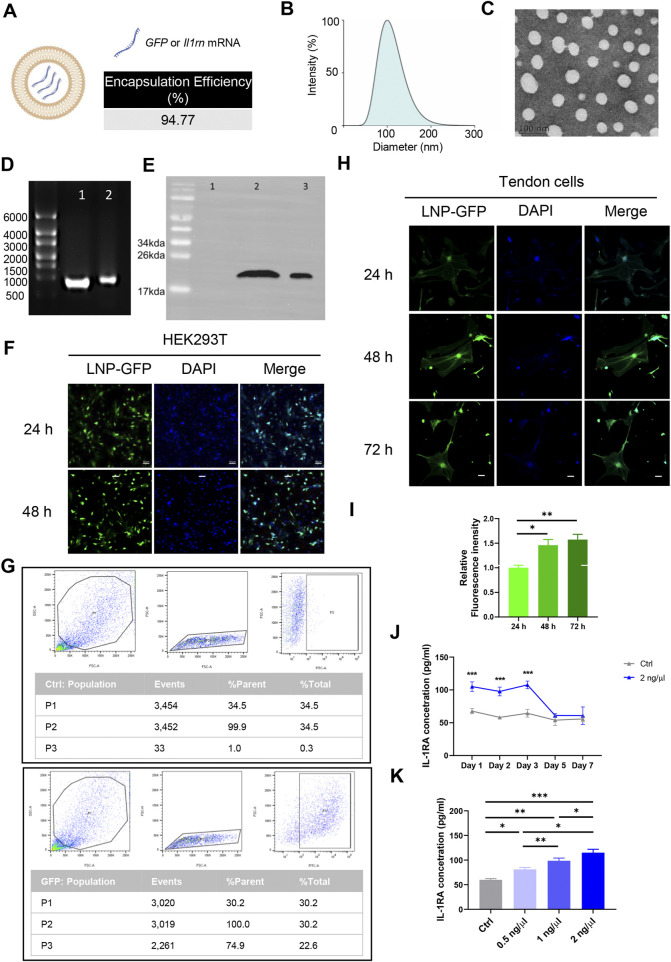
Assessment of the *in vitro* transfection efficiency of mRNA mediated by SM102 LNPs. **(A)** Schematic diagram of SM102 LNPs. **(B)** Dynamic light scattering distribution plot of SM102 LNPs-*Il1rn.* **(C)** Representative transmission electron microscopy image of SM102 LNPs-*Il1rn.* **(D)** Denaturing agarose gel electrophoresis of *Il1rn* mRNA before (lane 1) and after (lane 2) SM102 LNPs encapsulation. **(E)** Detection of IL-1RA protein in HEK293T cells treated with (lane 2) or without (lane 1) SM102 LNPs- *Il1rn* mRNA. Lane 3 was the positive control (Commercially available human IL-1RA protein) **(F)** Representative images showing the SM102 LNPs-mediated transfection of *GFP* mRNA in HEK293T cells. Scale bar: 50 μm. **(G)** Flow cytometry results of GFP positive cells for the control group (upper panel) and transfection group (Lower panel) at 48 h post-treatment. **(H)** Representative images showing the SM102 LNPs-mediated transfection of *GFP* mRNA in primary tendon stem cells. Scale bar: 50 μm. **(I)** Quantification of the fluorescence intensity of GFP from 24 h to 72 h post-transfection. **(J)** ELISA assay for detection of IL-1RA levels in the culture medium of tendon cells treated with 2 ng/μL SM102 LNPs- *Il1rn* mRNA from Day 1 to Day 7 post-transfection. **(K)** ELISA assay for detection of IL-1RA levels in the culture medium of tendon cells treated with different concentration of SM102 LNPs- *Il1rn* mRNA at 24 h post-transfection. *p < 0.05, **p < 0.01, ***p < 0.001; n = 3; All data are shown as the mean ± Standard Error of the Mean (SEM); Statistical significance was determined by one-way ANOVA with Fisher’s LSD test.

The physicochemical characterization and representative transmission electron microscopy images of SM102-based nano-liposome particles (LNPs)-mRNA was shown in Figures 2A–C. The denaturing agarose gel electrophoresis showed that the size of *Il1rn* mRNA before and after encapsulation was approximately 900 bp, which was consistent with the expected size (Figure 2D; Supplementary Figure S1). Co-incubation of SM102 LNPs- *Il1rn* mRNA with HEK293T cells for 48 h and the total protein was extracted to detect the expression of IL-1RA. The result indicated a protein band in the co-incubation group but not in the negative control (Figure 2E). The molecular weight of the band was around 20 kDa, which is comparable with the positive control (Figure 2E; Supplementary Figure S2). In addition, the delivery of *GFP* mRNA to HEK293T using SM102 LNPs enabled robust protein expression for at least 48 h (Figure 2F). To further evaluate the SM102 LNPs-mediated mRNA transfection efficiency on primary tendon stem cells, SM102 LNPs-*GFP* mRNA was used for co-incubation. At 48 h post-incubation, flow cytometry analysis showed that the proportion of GFP-positive cells in the treatment group was 74.9% (Figure 2G). Under fluorescence microscopy, significant GFP fluorescence signals were observed in the treatment group from 24 to 72 h post-transfection (Figure 2H). Quantification analysis indicated that the fluorescence intensity of GFP was significantly increased during the 3 days post transfection (Figure 2I). Similarly, ELISA assay showed that the expression of IL-1RA in the supernatant of the SM102 LNPs- *Il1rn* mRNA treatment group was significantly higher than that of the untreated group from 24 to 72 h (Figure 2J). These results implied the therapeutic time windows of the exogenous protein expression were around 72 h. Additionally, the transfected tendon cells could secrete IL-1RA in a dosage dependent manner (Figure 2K). The above results indicated that SM102 LNPs could efficiently transfect primary tendon stem cells and achieve functional protein expression for at least 72 h.

### 3.2 *In vitro* anti-inflammatory effect of IL-1RA in tendon cells

**FIGURE 3:**
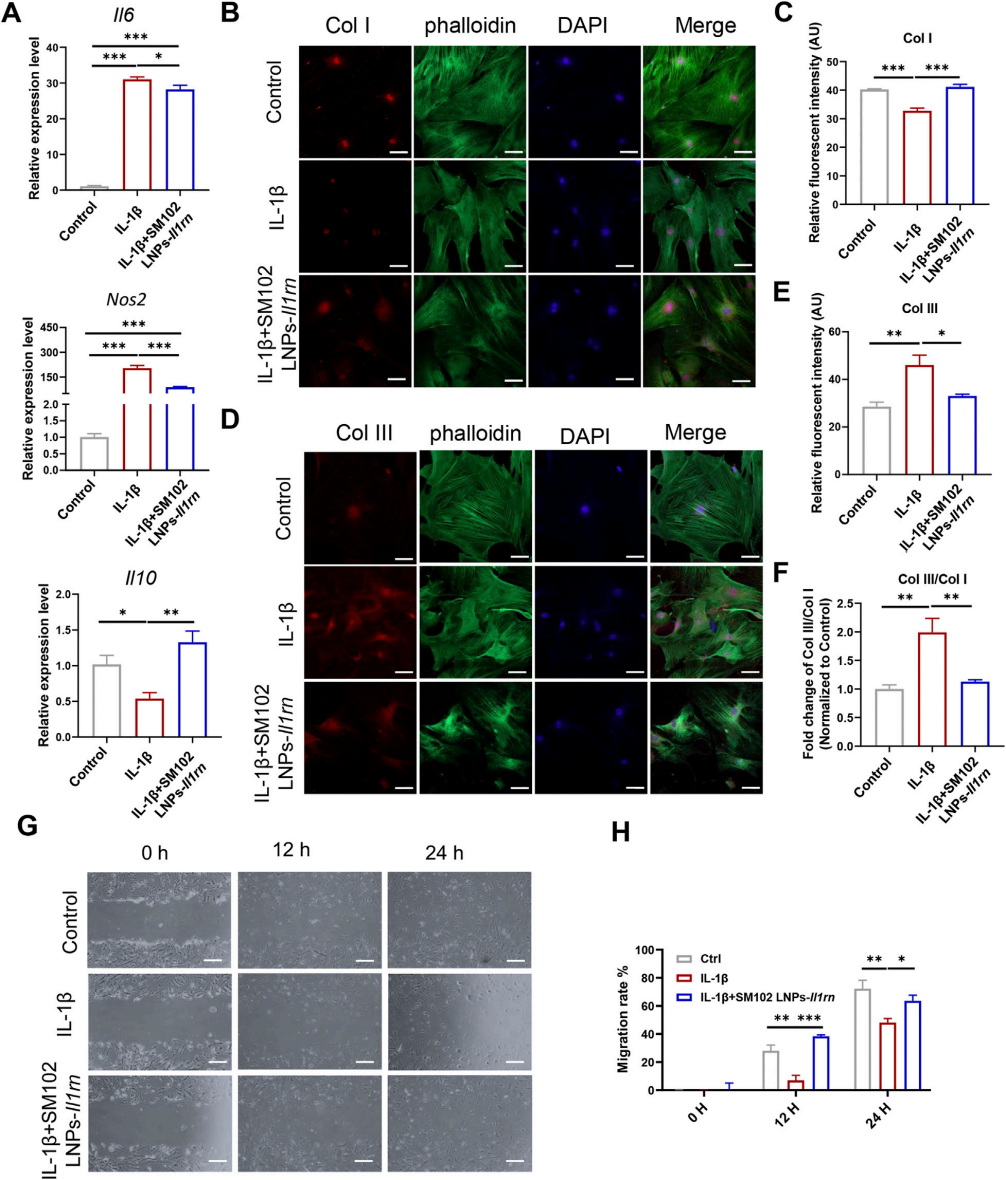
*In vitro* anti-inflammation property of IL-1RA on tendon stem cells. **(A)** qPCR results of *Il6* (upper), *Nos2* (middle) and *Il10* (Lower) in control, IL-1β and SM102 LNPs-*Il1rn* group. **(B)** Representative images showing the Col I expression in control, IL-1β, and IL-1β+SM102 LNPs-*Il1rn* group. Col I (Red); phalloidin (Green); Nucleus (Blue). Scale bar = 100 μm **(C)** Quantification analysis of Col I expression in control, IL-1β and IL-1β+ SM102 LNPs-*Il1rn* group. **(D)** Representative images showing the Col III expression in control, IL-1β and IL-1β+ SM102 LNPs-*Il1rn* group. Col III (Red); phalloidin (Green); Nucleus (Blue). Scale bar = 100 μm **(E)** Quantification analysis of Col III expression in control, IL-1β, and IL-1β+ SM102 LNPs-*Il1rn* group. **(F)** Quantification analysis of Col III/Col I expression in control, IL-1β, and IL-1β+ SM102 LNPs-*Il1rn* group. **(G)** Representative microscopic images showing the scratch closure in control, IL-1β, and IL-1β+ SM102 LNPs-*Il1rn* group at 0 h, 12 h, and 24 h. Scale bar = 200 μm **(H)** Quantification of scratch closure in different groups at 0 h, 12 h, and 24 h *p < 0.05, **p < 0.01, ***p < 0.001; n = 3; All data are shown as the mean ± Standard Error of the Mean (SEM); Statistical significance was determined by one-way ANOVA with Fisher’s LSD test **(A,C,E)**; Statistical significance was determined by two-way ANOVA with Fisher’s LSD test **(G)**.

Inflammatory responses alter the tendon microenvironment (Chisari et al., 2020). Existing evidence indicates that exogenous supplementation of IL-1β will increase the expression of a series of genes such as Interleukin 6 (*Il10*) in tendon cells (Tsuzaki et al., 2003; Vinhas et al., 2020). To verify the anti-inflammatory effect of IL-1RA, tendon stem cells transfected with or without SM102 LNPs- *Il1rn* mRNA were stimulated with 10 ng/mL IL-1β for 24 h qPCR results showed that IL-1β significantly upregulated *Il6*, inducible Nitric Oxide Synthase 2 (*Nos2*) and downregulated Interleukin 10 *(Il10)* (Figure 3A). Overexpression of IL-1RA could partially reverse the abnormal changes of the inflammatory-related genes (Figure 3A). Normal tendon ECM is mainly composed of collagen I (Col1), while in tendon lesions, the deposition of disordered collagen III (Col3) significantly increases, and the imbalance of Col3/Col1 ratio becomes a characteristic pathological marker (Tu et al., 2023; Franchi et al., 2007). Immunofluorescence staining confirmed that IL-1β treatment significantly decreased the Col I expression, increased the Col III expression and Col III/Col I in tendon stem cells, while overexpression of IL-1RA in tendon stem cells restored their expression levels comparable to the normal mice (Figures 3B–F). In addition, the effects of IL-1RA on tendon cell migration under IL-1β stimulation were evaluated by cell scratch assays. Scratch assays indicated that IL-1β significantly reduced migration of tendon stem cells at 12 and 24 h, while overexpression of IL-1RA significantly inhibited this effect (Figures 3G,H).

These results proved that IL-1RA can regulate inflammatory levels, improve ECM metabolic imbalance, and inhibit abnormal cell behaviors induced by IL-1β.

### 3.3 Therapeutic efficacy of SM102 LNPs- *Il1rn* mRNA in tendinopathy mice model

Before evaluating the therapeutic efficacy of SM102 LNPs-*Il1rn* mRNA, we first injected Luciferase mRNA into the tendon region of mice to determine whether SM102 LNPs could effectively deliver mRNA *in vivo*. Imaging was performed at 1-, 3-, and 7-day post-injection (Supplementary Figure S3A). Fluorescent signals indicated the successful delivery of mRNA to the tendon tissue by SM102 LNPs (Supplementary Figure S3A). Quantification analysis confirmed significantly higher signal levels in injected mice compared to control mice, persisting for 3 days, which is consistent with the *in vitro* data (Supplementary Figure S3B). To investigate the biodistribution of luciferase protein, major organs were dissected for imaging at 24 h post-injection. Luciferase signals were detectable in typical storage organs such as the liver and spleen (Rosanaly et al., 2025). However, the signal intensity in these organs was significantly lower, accounting for approximately 10% (liver) and 0.5% (spleen) of that observed in tendon tissue (1.7E+05) (Supplementary Figure S3C).

**FIGURE 4:**
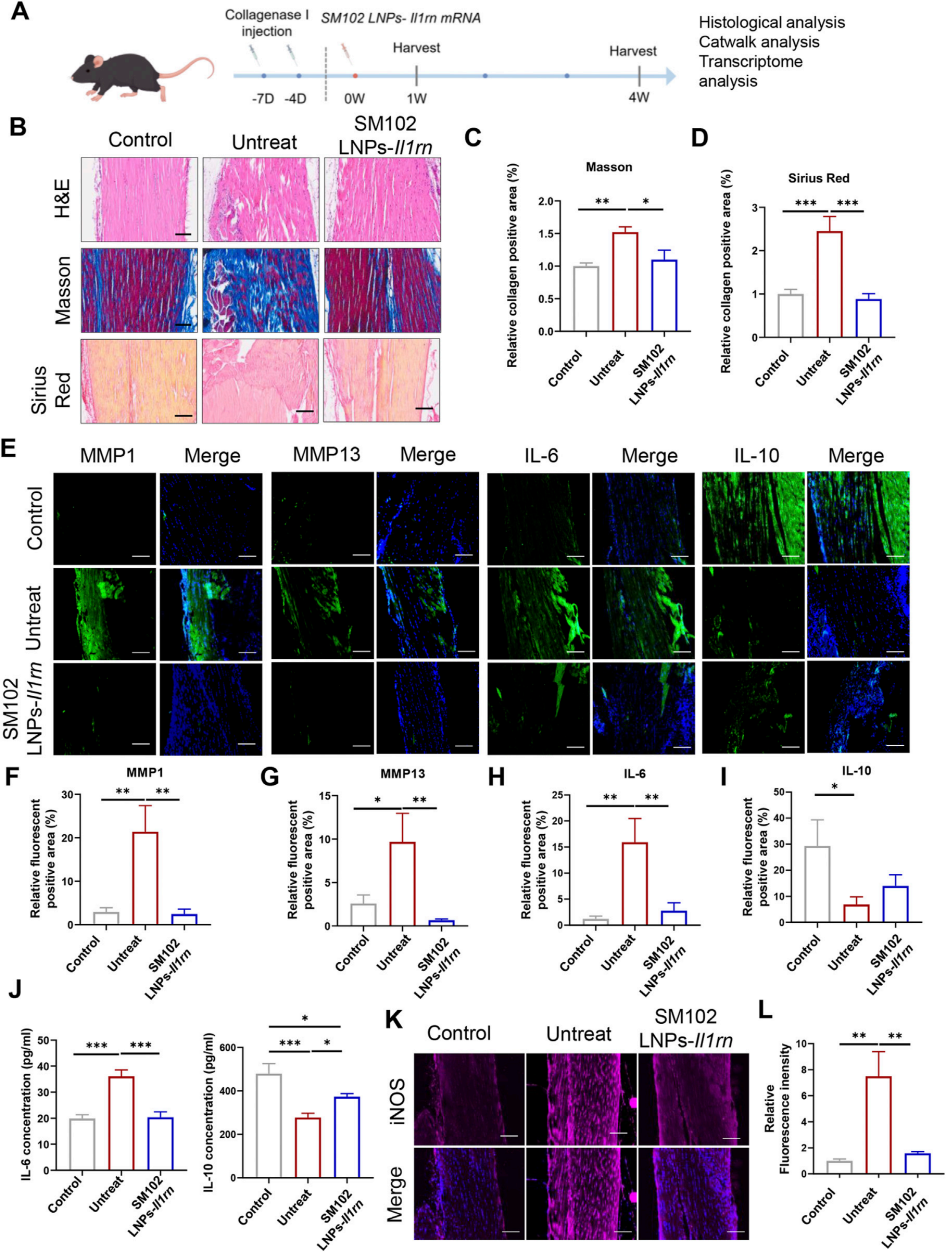
*In vivo* therapeutic effects of SM102 LNPs- *Il1rn* mRNA on tendon tissues at week 1. **(A)** Scheme of animal experiment design (By Figdraw). **(B)** Representative images of H&E staining (upper panel), Masson’s trichrome staining (middle panel), and Sirius red staining (lower panel) of tendons from different groups in the first week postoperatively. Scale bar = 100 µm **(C,D)** Quantification analysis of Masson’s trichrome staining **(C)** and Sirius red staining **(D)**. **(E)** Representative images of immunofluorescent staining of MMP1, MMP13, IL-6, and IL-10 of sections of each group in the first week postoperatively. Scale bar = 100 µm **(F–I)** Quantification of MMP1, MMP13, IL-6, and IL-10 expression in different groups. **(J)** Elisa assay for detection of IL-6 (Left) and IL-10 (Right) expression level in tendon tissue from different groups. **(K)** Representative images of immunofluorescent staining of iNOS of sections of each group in the first week postoperatively. Scale bar = 100 µm **(L)** Quantification of iNOS expression in different groups. *p < 0.05; **p < 0.01; ***p < 0.001; n = 5; All data are shown as the mean ± Standard Error of the Mean (SEM). Statistical significance was determined by one-way ANOVA with Fisher’s LSD test.

A murine model of tendinopathy was established via type I collagenase injection to evaluate the anti-inflammatory efficacy of SM102 LNPs-IL-1RA *in vivo* (Figure 4A). Histopathological analysis at 1-week post-treatment revealed that untreated tendons exhibited disorganized extracellular matrix (ECM) architecture, whereas SM102 LNPs- *Il1rn* mRNA treatment restored regular fiber alignment (Figure 4B). Masson’s trichrome and Sirius red staining demonstrated excessive collagen deposition in the untreated group, which was significantly attenuated by SM102 LNPs-Il1rn mRNA treatment (Figures 4C,D). Immunofluorescence analysis further indicated elevated expression of pro-inflammatory IL-6, matrix-degrading enzymes MMP1/MMP13, and suppressed IL-10 levels in untreated tendons, consistent with sustained inflammation and ECM catabolism. These pathological markers were normalized to near-physiological levels in the treatment group (Figures 4E–I). The ELISA analysis revealed a comparable expression profile for IL-6 and IL-10 in tendon tissues to the immunofluorescence results (Figure 4J). Furthermore, IL-1RA treatment significantly reduced iNOS expression in the tendon tissue of tendinopathy mice (Figures 4K,L).

**FIGURE 5:**
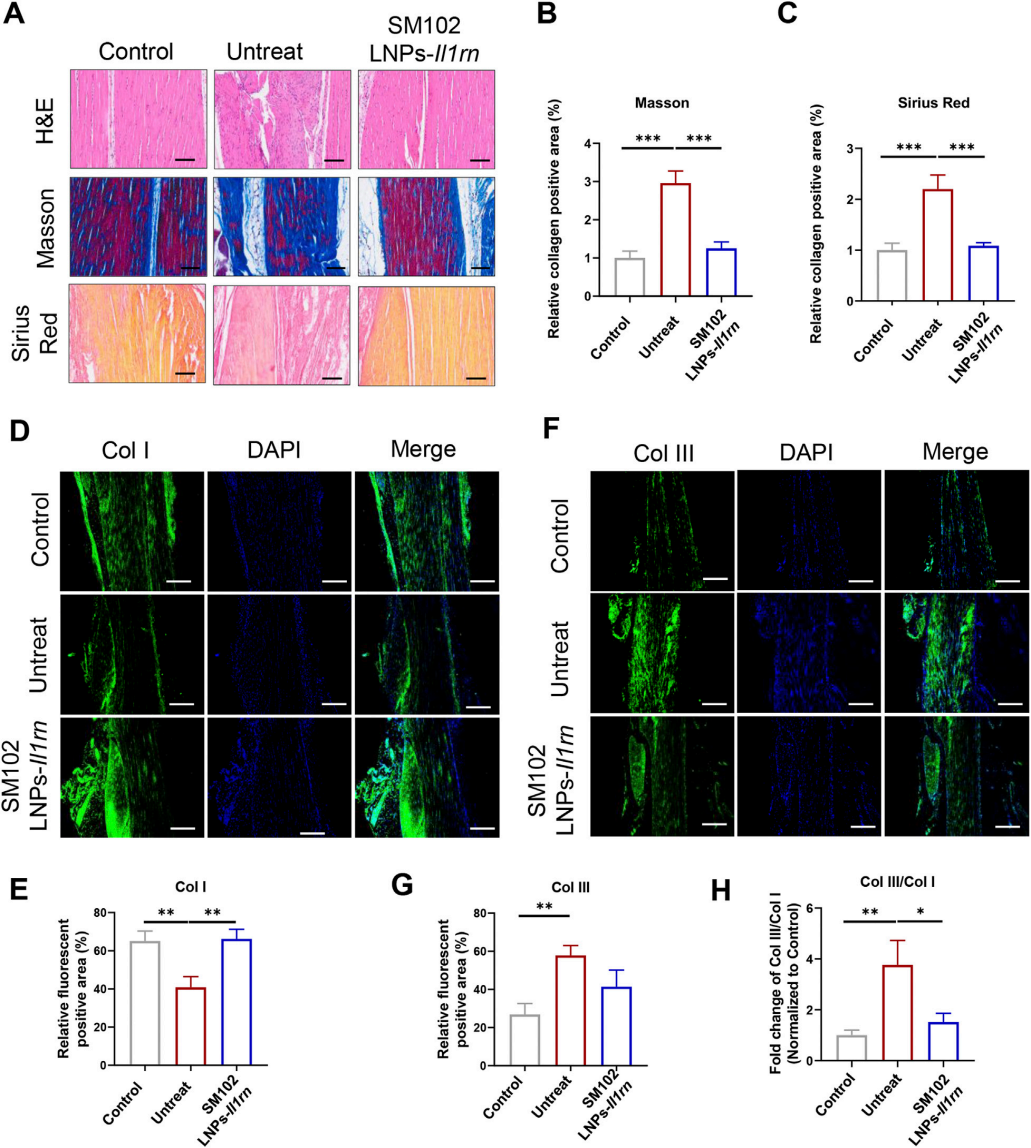
*In vivo* therapeutic effects of SM102 LNPs- *Il1rn* mRNA on tendon tissues at week 4. **(A)** Representative images of H&E staining (upper panel), Masson’s trichrome staining (middle panel), and Sirius red staining (lower panel) of tendons from different groups in the fourth week postoperatively. Scale bar = 100 µm **(B,C)** Quantification analysis of Masson’s trichrome staining **(B)** and Sirius red staining **(C)**. **(D)** Representative images of immunofluorescent staining of Col I of sections of each group in the fourth week postoperatively. Scale bar = 100 µm **(E)** Quantification Col I expression in different groups. **(F)** Representative images of immunofluorescent staining of Col III of sections of each group in the fourth week postoperatively. Scale bar = 100 µm **(G)** Quantification Col III expression in different groups. **(H)** Quantification Col III/Col I expression in different groups. *p < 0.05; **p < 0.01; ***p < 0.001; n = 5; All data are shown as the mean ± Standard Error of the Mean (SEM). Statistical significance was determined by one-way ANOVA with Fisher’s LSD test.

At 4 weeks post-treatment, untreated tendons persistently displayed disordered collagen architecture and hyperdeposition, while M102 LNPs- *Il1rn* mRNA- treated mice exhibited mitigated pathological features (Figures 5A–C). Quantitative immunofluorescence analysis of collagen subtypes revealed a marked decrease in Col I and an increase in Col III and the Col III/Col I ratio in tendinopathy mice (Figures 5D–H). In contrast, SM102 LNPs-Il1rn mRNA treatment reversed this imbalance, significantly downregulating Col I, upregulating Col III, and increasing the Col III/Col I ratio compared to the untreated group (Figures 5D–H).

**FIGURE 6:**
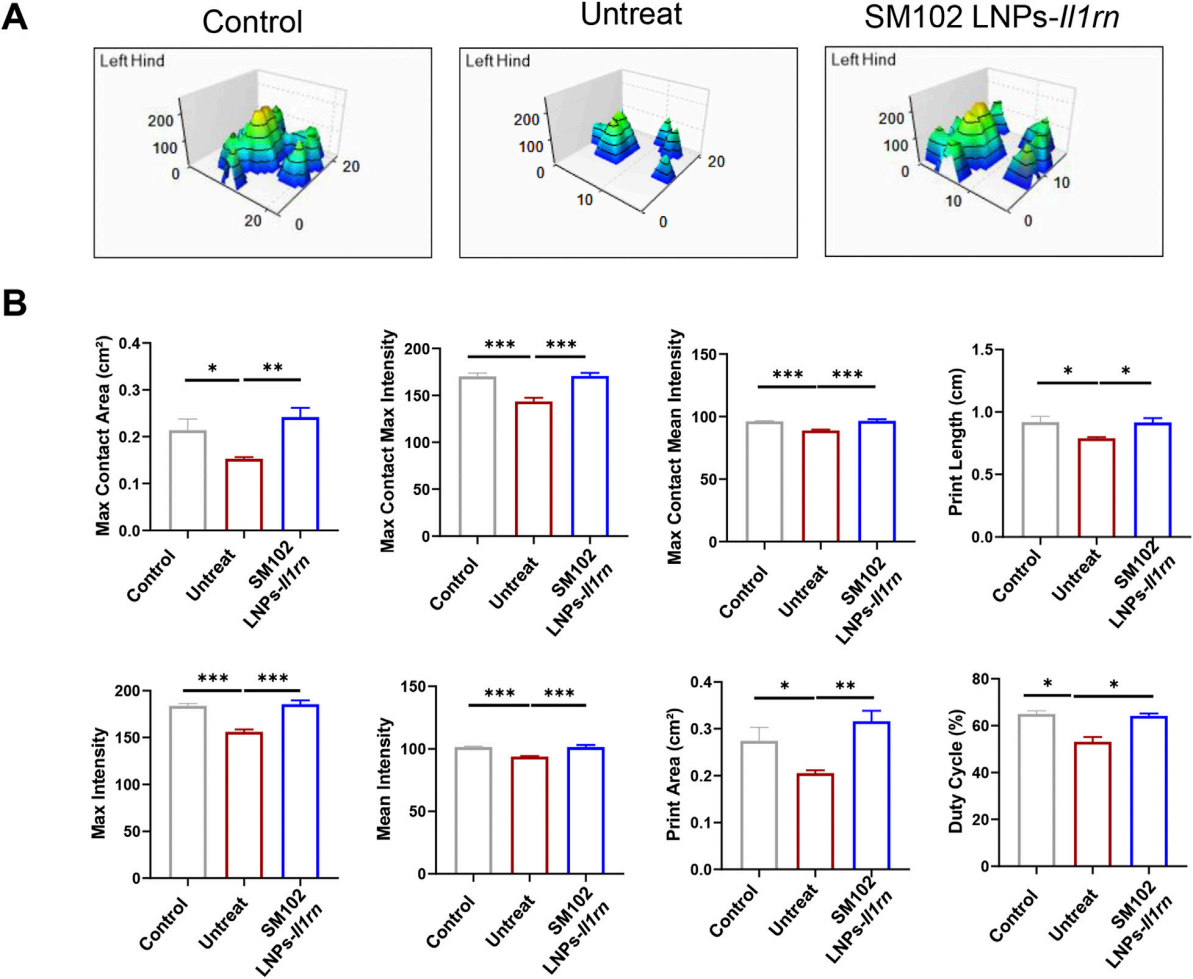
Catwalk for gait analysis after SM102 LNPs- *Il1rn* mRNA treatment. **(A)** Representative 3D footprint intensity images of control (left panel), untreated (middle panel), and treated (right panel) groups at 4 weeks after treatment. **(B)** The quantification analysis of the catwalk for gait analysis. *p < 0.05, **p < 0.01, ***p < 0.001; n = 5; All data are shown as the mean ± Standard Error of the Mean (SEM). Statistical significance was determined by one-way ANOVA with Fisher’s LSD test.

Functional recovery was assessed via 3D gait analysis. The treatment group displayed a footprint intensity map resembling healthy controls (Figure 6A), with quantitative analysis confirming significant improvement in gait patterns and near-restoration to control levels (Figure 6B). For instance, tendinopathy mice exhibited significantly smaller contact areas and lower intensity values than the control group (Figure 6B), indicating pain-induced unloading of the affected limb to minimize mechanical pressure. Conversely, the restoration of these parameters to near-normal levels in the IL-1RA treatment group demonstrates that local IL-1 blockade effectively reverses tendon damage-induced dysfunction. These results demonstrate that SM102 LNPs-IL-1RA alleviates tendinopathy by resolving inflammation, normalizing collagen composition, and restoring motor function.

### 3.4 Transcriptome analysis on SM102 LNPs- *Il1rn* mRNA-mediated therapeutic effect in tendinopathy mice model

**FIGURE 7:**
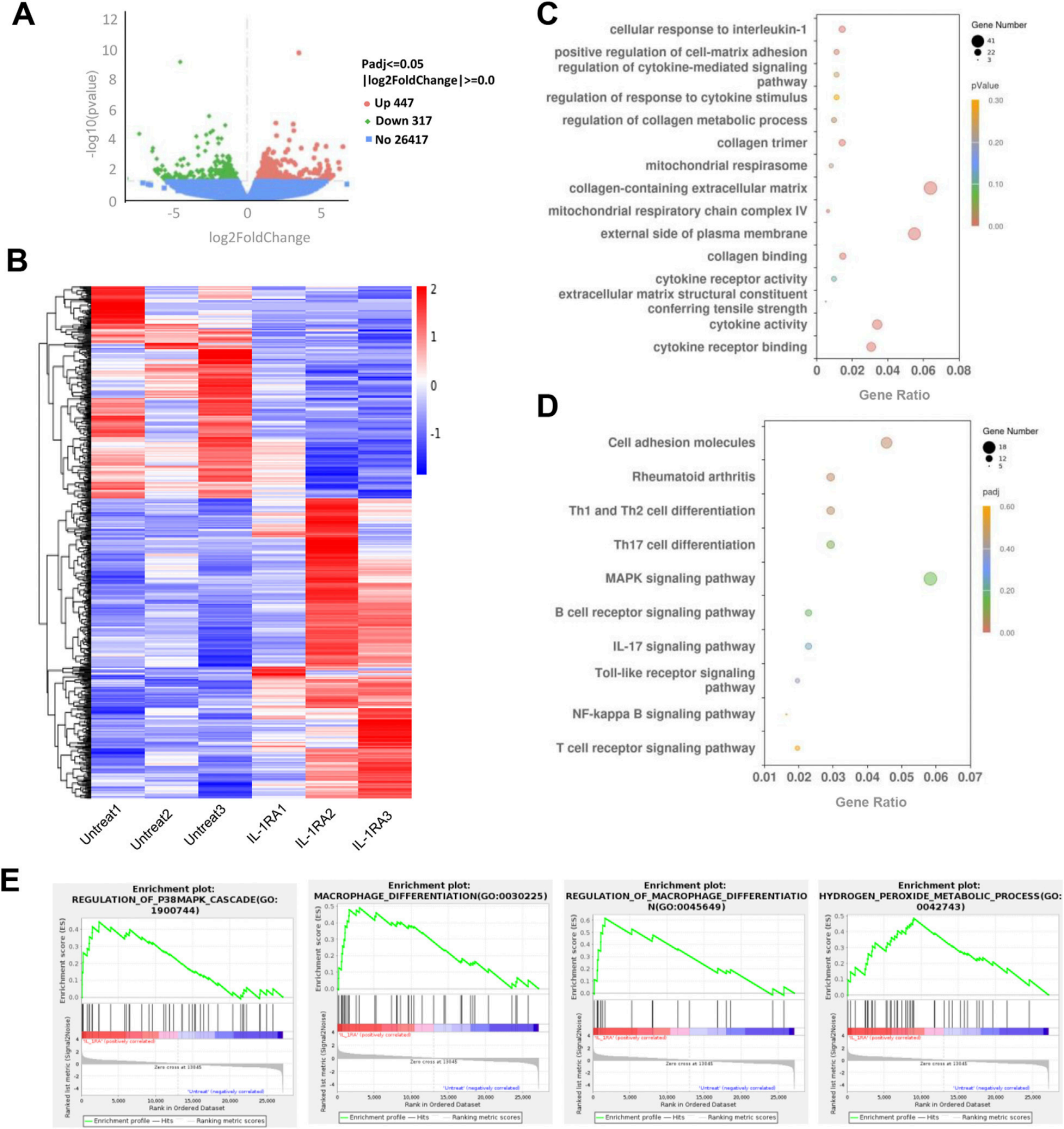
Transcriptome analysis of tendon tissues between the untreated and treated groups. **(A)** Volcano plot of gene expression (Treated versus Untreated; fold change, ≥1; q value <0.05). **(B)** Heat map of differentiated expression genes. **(C)** GO analysis of differentially expressed genes. **(D)** KEGG analysis of differentially expressed genes in the treatment group. **(E)** GSEA plot of the genes associated with P38MAPK signaling, microphage differentiation, and hydrogen peroxide metabolic process. FDR <0.25.

RNA sequencing analysis of mouse tendon tissues revealed 447 downregulated genes and 317 upregulated genes (|Log2FoldChange| ≥1, Padj ≤0.05) in the SM102 LNPs-Il1rn mRNA group compared to controls (Figure 7A). Differential gene expression analysis (Figure 7B) and Gene Ontology (GO) enrichment (Figure 7E) demonstrated that SM102 LNPs-Il1rn mRNA treatment specifically modulated cellular responses to IL-1 and extracellular matrix (ECM) remodeling (*e.g.*, regulation of collagen metabolic processes and collagen trimer formation), suggesting that the therapy suppresses inflammation by blocking IL-1 signaling while promoting collagen synthesis and ECM repair. Kyoto Encyclopedia of Genes and Genomes (KEGG) pathway analysis further supported these findings, showing regulation of inflammatory signaling pathways such as NF-κB and IL-17 signaling in the treatment group (Figures 7C, D). The enrichment of the MAPK signaling pathway aligned with Gene Set Enrichment Analysis (GSEA) results (Figure 7E). In addition, GSEA analysis also found enhanced macrophage differentiation and hydrogen peroxide metabolism in treatment groups (Figure 7E).

Collectively, these transcriptomic findings suggest that SM102 LNPs-*Il1rn* mRNA exerts dual therapeutic effects in tendinitis by targeting IL-1 signaling—simultaneously inhibiting inflammatory cascades and activating collagen-driven ECM restoration. The coordinated modulation of MAPK signaling, macrophage polarization, and H_2_O_2_ metabolism may further contribute to reestablishing tissue homeostasis.

### 3.5 Body safety evaluation

**FIGURE 8:**
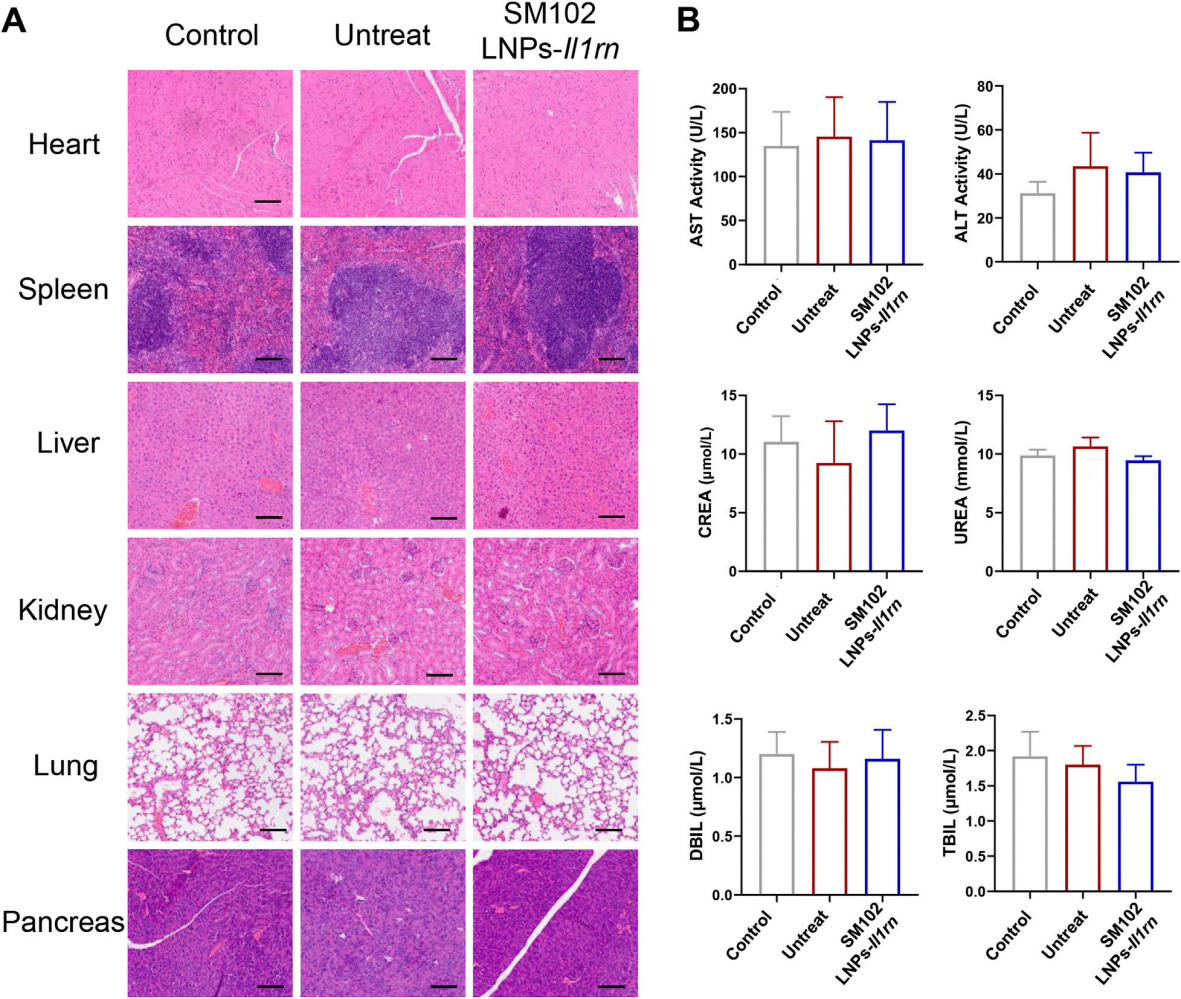
Evaluation of Biosafety of SM102 LNPs- *Il1rn* mRNA treatment. **(A)** H&E-stained sections of rat heart, spleen, liver, kidney, lung, and pancreas from Control, Untreated, and Treated groups after a 4-week intervention. Scale bar = 100 µm **(B)** Biochemical analysis of serum markers indicating organ function in different groups. The panels display levels of AST, ALT, CREA, UREA, DBIL, and TBIL. *p < 0.05; **p < 0.01; ***p < 0.001; n = 5; All data are shown as the mean ± Standard Error of the Mean (SEM). Statistical significance was determined by one-way ANOVA with Fisher’s LSD test.

The safety of SM102 LNPs- *Il1rn* mRNA was evaluated by H&E staining of major organs and serum biochemical indicators (Figure 8A). Histological analysis showed that the structures of organs such as the heart, liver, spleen, lung, and kidney in the treatment group were intact, and no degenerative changes or inflammatory cell infiltration were observed. Also, there was no significant difference in serum biochemical parameters (such as liver and kidney function indicators) between the treatment group and the control group, confirming the good biological safety of SM102 LNPs- *Il1rn* mRNA treatment (Figure 8B).

## 4 Discussion

**FIGURE 9:**
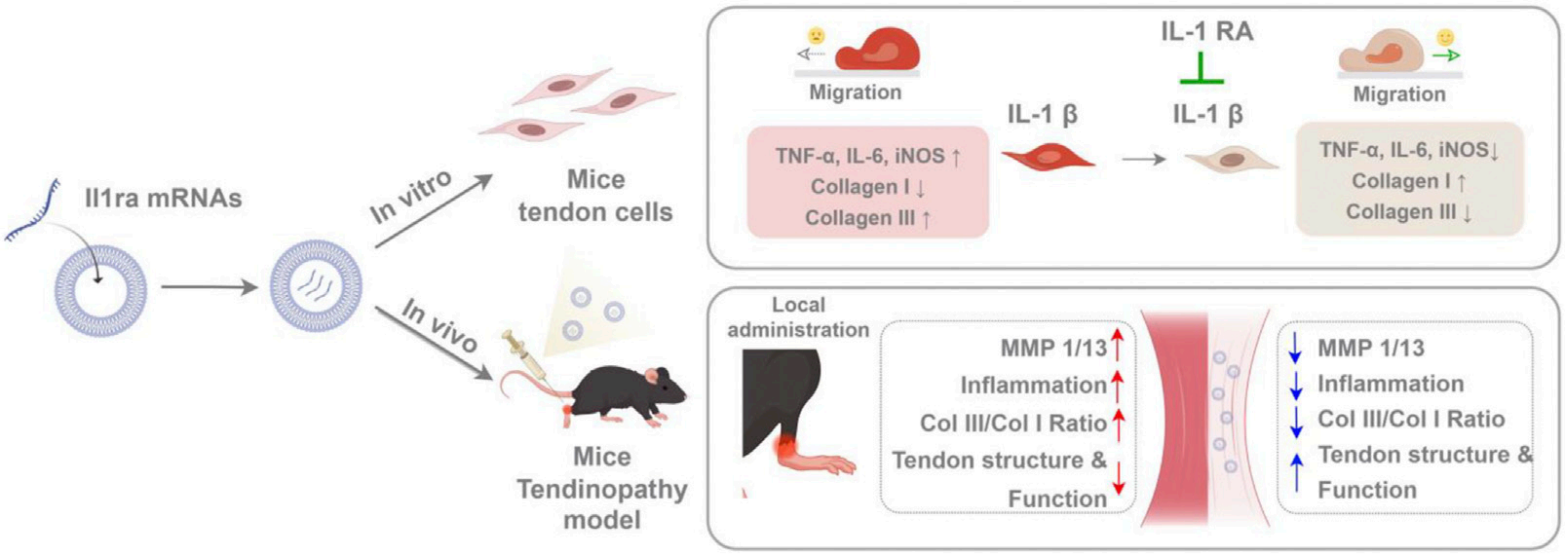
Schematic illustrating SM102 LNPs- *Il1rn* mRNA-mediated therapeutic mechanisms against tendinopathy through coordinated modulation of inflammation, MMPs expression, collagen synthesis, and tenocyte migration (By Figdraw).

Tendinopathy is a disease characterized by chronic inflammation and matrix degeneration as its core pathological features (Lipman et al., 2018; Rees et al., 2006). There were two challenges in the treatment: how to effectively block matrix degradation mediated by pro-inflammatory signals and achieve precise drug delivery and sustained action of therapeutic drugs. In this study, SM102 lipid nanoparticles (LNPs) were used for the local delivery of *Il1rn* mRNA to the tendon tissue. We systematically investigated the anti-inflammatory, pro-healing effects and biological safety of SM102 LNPs- *Il1rn* mRNA *in vitro* and *in vivo* (Figure 9).

The low vascularization characteristic of tendon tissue limits the efficiency of drug delivery (Loiacono et al., 2019). Although various delivery systems have been developed for local treatment strategies for tendon diseases, their clinical translation still faces challenges (Adjei-Sowah et al., 2024; Han et al., 2024; Beldjilali-Labro et al., 2018). The success of the COVID-19 vaccine has verified the feasibility of lipid nanoparticles for delivering mRNA. The application of this technology in musculoskeletal diseases (especially tendon diseases) is in the exploration stage (Sun et al., 2022; López-Cerdá et al., 2024; Parchi et al., 2016; Huang et al., 2025). Ionizable lipid SM102 undergoes protonation in the acidic endosome environment, promoting mRNA escape to the cytoplasm, thereby achieving efficient expression (Schoenmaker et al., 2021; Hassett et al., 2019; Maier et al., 2013; Corbett et al., 2020). Our study found that SM102-LNPs can efficiently transfect primary tendon stem cells. Notably, our experiments demonstrated that SM102 LNPs-mediated delivery of mRNA achieved sustained expression of exogenous IL-1RA protein for approximately 72 h in tendon tissue. In contrast, literature reports indicate that subcutaneously administered recombinant proteins (molecular weight range: 23–149 kDa) are largely cleared from the injection site within 24 h, with clearance rates inversely correlating with molecular weight (Wu et al., 2012). These data support that mRNA therapy provides an extended therapeutic window compared to protein therapy. Our experiments demonstrated a clear dose-response relationship in SM102 LNPs-mediated exogenous gene expression, indicating that treatment effects can be precisely controlled by dose adjustment. This finding provides critical evidence for developing customized clinical therapies.

IL-1β is one of the key factors contributing to the development of tendon disorders (Morita et al., 2017). It drives the expression of pro-inflammatory factors and matrix metalloproteinases, leading to collagen degradation and fibrosis (Morita et al., 2017). To evaluate the anti-inflammatory function of SM 102 LNPs- *Il1rn* mRNA. Primary tendon stem cells were incubated with IL-1β. And the results illustrated that the external IL-1β could stimulate the expression of IL-6 and TNF-α in tendon stem cells, which is consistent with a previous study (Vinhas et al., 2020). In addition, we also found that IL-1β could induce iNOS expression in primary tendon stem cells, similar to skeletal myoblasts (Adams et al., 2002). *In vitro* application of SM 102 LNPs- *Il1rn* mRNA on tendon stem cells reversed the activation of these target genes. Also, our findings demonstrated that IL-1RA reduced the IL-1β induced imbalance of Col III/Col I and the inhibition of tendon cell migration. These *in vitro* data helped to explain the therapeutic efficacy of SM 102 LNPs- *Il1rn* mRNA in the mouse model of tendinopathy.

Inflammation is a natural response to injury, and it plays a central role in the development of tendon disorders. Previous studies found that interleukin-1 (IL-1) significantly increased in the stress-shielded achilles tendons of rats, and the application of IL-1RA could prevent the morphological deterioration of tendons via inhibiting the elevation of MMP1, and improving collagen metabolism (Ma et al., 2012). Similarly, our experiments also demonstrated that the single injection of SM102 LNPs- *Il1rn* mRNA could modulate the inflammatory-matrix-degrading axis by increasing anti-inflammatory factors (e.g., IL-10), suppressing pro-inflammatory cytokines (e.g., IL-6), reducing matrix-degrading enzymes (e.g., MMP1/13) and downregulating Inflammatory marker iNOS within the first week post-treatment. At 4 weeks post-injection, treated mice exhibited significantly improved tendon fiber alignment compared to the tendinitis control group, with collagen I and III expression and the Col III/I ratio restored to baseline levels, a recovery pattern correlated with enhanced motor function in gait analyses.

The RNA-seq analysis further emphasized the regulating effect of SM102 LNPs- *Il1rn* mRNA in inflammation, ECM homeostasis, and collagen synthesis. It is worth noting that existing evidence indicates that the anabolic effects (such as the downregulation of type I collagen and the upregulation of MMP) of IL-1β in human tendon fibroblasts were mediated via the p38 MAPK signaling cascade (Chen et al., 2024). In our study, the relationship between p38 MAPK signaling and the effect of SM102 LNPs- *Il1rn* mRNA was also confirmed by KEGG pathway analysis and GSEA analysis.

In conclusion, this study has demonstrated that the *Il1rn* mRNA therapy based on SM102-LNPs can effectively inhibit inflammation, reverse matrix degradation, and promote functional recovery through efficient delivery, transient expression, and multi-target regulation mechanisms (Figure 9). Its modular design supports the flexible substitution of other therapeutic mRNAs (such as anti-fibrotic or pro-angiogenic factors), providing a new strategy for the precise treatment of tendon diseases and other degenerative disorders of soft tissues. With the optimization of mRNA delivery technology and the advancement of clinical translation, this method is expected to become a new alternative to traditional surgery and hormone therapy.

## Data Availability

The datasets presented in this study can be found in online repositories. The names of the repository/repositories and accession number(s) can be found below: https://www.ncbi.nlm.nih.gov/, PRJNA1264510.
