# Supplementary material for: Anti-inflammatory therapy for tendinopathy using Il1rn mRNA encapsulated in SM102 lipid nanoparticles
Source: Front Bioeng Biotechnol. 2025 Aug 12;13:1641236. doi: 10.3389/fbioe.2025.1641236 (PMC12378938; doi:10.3389/fbioe.2025.1641236)
Supplement: Supplementary file 1 [file DataSheet1.pdf]

Supplementary Material

Yuan Zhang [1,2†] , Xu Li [3†], Hao Li [4], Ruiyang Zhang [4], Ti Zhang [5], Talante Juma [1], Yongfei Zhou [6] , Quanyi Guo [4*] , Hui Zhao [2*], Yongping Cao [1*]

[1] Department of Orthopedics, Peking University First Hospital, Beijing, China.

[2] Key Laboratory for Regenerative Medicine of the Ministry of Education of China, School of Biomedical Sciences, Faculty of Medicine, The Chinese University of Hong Kong, Shatin, Hong Kong SAR, People's Republic of China.

[3] National Center for Orthopaedics, Beijing Jishuitan Hospital, Capital Medical University, Beijing, China.

[4] Institute of Orthopedics, the First Medical Center, Chinese PLA General Hospital; Beijing Key Lab of Regenerative Medicine in Orthopedics; Key Laboratory of Musculoskeletal Trauma & War Injuries PLA; No. 28 Fuxing Road, Haidian District, Beijing 100853, China.

[5] Department of Orthopedics, Peking University Third Hospital, 49 North Garden Road, Haidian District, Beijing 100191, People’s Republic of China.

[6] Institute of Hemu Biotechnology, Beijing Hemu Biotechnology Co., Ltd, Beijing, 102206, China.

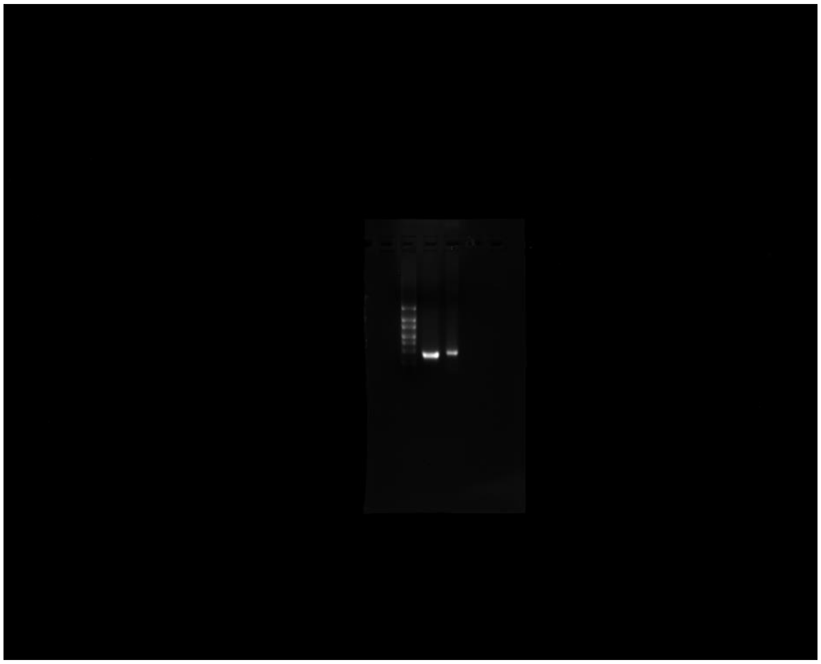

Supplementary Figure 1. Original image of Figure 2D**.**

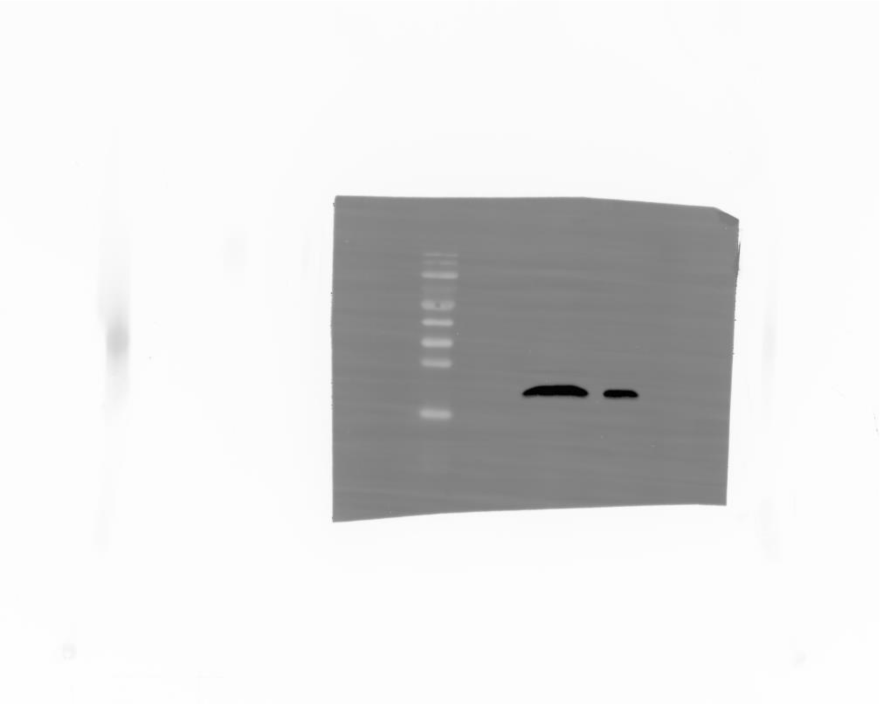

Supplementary Figure 2. Original image of Figure 2E.

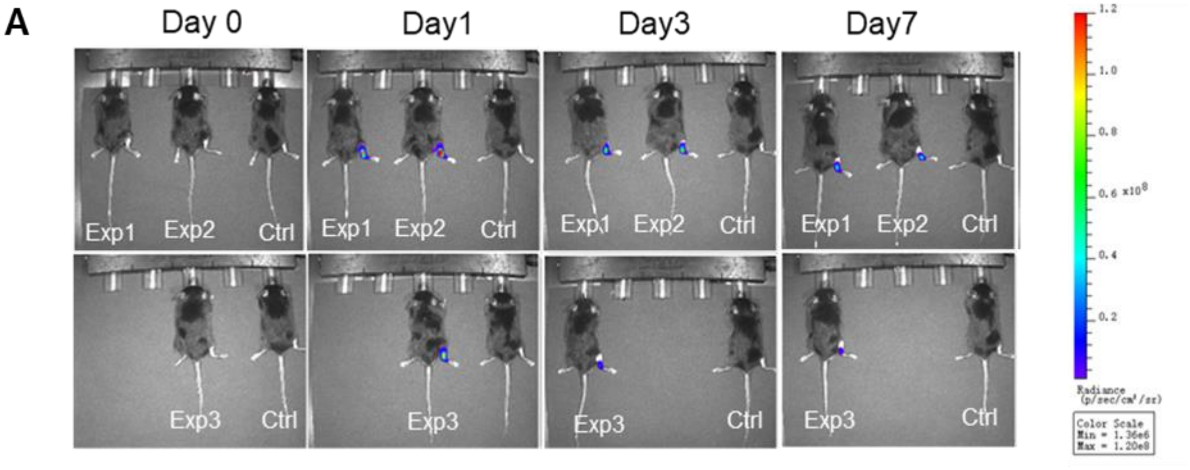

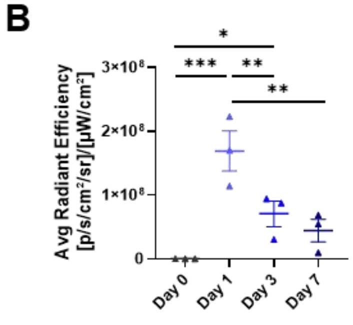

C

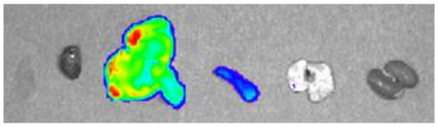

Tissue	Average Radiant Efficiency
Heart	1.75E+05
Liver	1.74E+07
Spleen	8.17E+05
Lung	7.93E+04
Kidney	4.05E+04

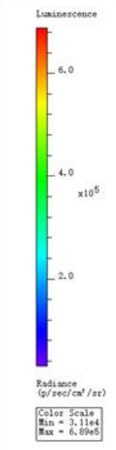

Supplementary Figure 3. *In vivo* imaging of mice with mRNA or protein injection. (A) *In vivo* fluorescence images (Left) of the tendon after subcutaneous injection of SM102 LNPs- *Il1rn* mRNA (Exp1 to Exp3) (B) the average radiance [p/s/cm^2/sr] was measured at Day 0, 1, 3, and 7 after injection. *$p < 0.05$; ** $p < 0.01$; *** $p < 0.001$; n=3; All data are shown as the mean ± Standard Error of the Mean (SEM). Statistical significance was determined by one-way ANOVA with Fisher's LSD test. (C) *In vivo* fluorescence images of major organs at 24 hours post-injection of SM102 LNPs encapsulating IL1RN mRNA.
